# Colorectal cancer risk genes are functionally enriched in regulatory pathways

**DOI:** 10.1038/srep25347

**Published:** 2016-05-05

**Authors:** Xi Lu, Mingming Cao, Su Han, Youlin Yang, Jin Zhou

**Affiliations:** 1 Department of Gastroenterology, The First Affiliated Hospital of Harbin Medical University, No. 23 You zheng Street Nan Gang District, Harbin, Heilongjiang 150001, China; 2 Department of Endocrinology, The First Affiliated Hospital of Harbin Medical University, No. 23 You zheng Street Nan Gang District, Harbin, Heilongjiang 150001, China; 3 Department of Parasitology, Harbin Medical University, Harbin, Heilongjiang 150081, China; 4 Department of Hematology, The First Affiliated Hospital of Harbin Medical University, No. 23 You zheng Street Nan Gang District, Harbin, Heilongjiang 150001, China

## Abstract

Colorectal cancer (CRC) is a common complex disease caused by the combination of genetic variants and environmental factors. Genome-wide association studies (GWAS) have been performed and reported some novel CRC susceptibility variants. However, the potential genetic mechanisms for newly identified CRC susceptibility variants are still unclear. Here, we selected 85 CRC susceptibility variants with suggestive association *P* < 1.00E-05 from the National Human Genome Research Institute GWAS catalog. To investigate the underlying genetic pathways where these newly identified CRC susceptibility genes are significantly enriched, we conducted a functional annotation. Using two kinds of SNP to gene mapping methods including the nearest upstream and downstream gene method and the ProxyGeneLD, we got 128 unique CRC susceptibility genes. We then conducted a pathway analysis in GO database using the corresponding 128 genes. We identified 44 GO categories, 17 of which are regulatory pathways. We believe that our results may provide further insight into the underlying genetic mechanisms for these newly identified CRC susceptibility variants.

Colorectal cancer (CRC) is the third most common form of cancer and the second leading cause of cancer-related death in the western world and^1^,^2^. CRC is a leading cause of cancer-related deaths in the United States, and its lifetime risk in the United States is about 7%^1^,^3^. CRC is a common complex disease caused by the combination of genetic variants and environmental factors^1^. Genome-wide association studies (GWAS) are considered to be new and power approaches to detect the genetic variants of human complex diseases. Recently, GWAS have been performed and reported some novel CRC susceptibility single nucleotide polymorphisms (SNPs) with genome-wide significance (*P* < 5.00E-08), and these SNPs have been repliciated by meta-anaysis methods^4^,^5^,^6^,^7^,^8^,^9^,^10^,^11^,^12^,^13^.

In 2012, Loo *et al.* conducted a cis-expression quantitative trait loci (cis-eQTLs) analysis to investigate the possible regulatory functions of 19 CRC risk variants on the expression of neighboring genes (<2 Mb up- or down-stream)^14^. They identified three variants including rs10795668, rs4444235 and rs9929218 to be significantly associated with expression levels of nearby genes^14^. In 2014, Closa *et al.* analyzed the association between 26 CRC SNPs and the expression of genes within a 2 Mb region (cis-eQTLs) using 47 healthy colonic mucosa tissues and 97 normal mucosa tissues adjacent to colon cancer, and 97 paired tumor tissues^15^. Using Bonferroni correction, they identified three significant cis-eQTLs including rs3802842 in 11q23.1 associated with the expression of C11orf53, COLCA1 and COLCA2; rs7136702 in 12q13.12 associated with the expression of DIP2B and rs5934683 in Xp22.3 associated with the expression of SHROOM2 and GPR143. Closa *et al.* also reported other SNPs including rs7130173 for 11q23.1 and rs61927768 for 12q13.12, which are in linkage disequilibrium (LD) with rs3802842 and rs7136702, and are more strongly associated with the expression of the identified genes and are better functional candidates. In 2014, Yao *et al.* select 25 CRC SNPs, and test the hypothesis that the CRC SNPs and/or correlated SNPs are in elements that regulate gene expression^3^. They identified 23 promoters, 28 enhancers, and 66 putative target genes of the risk-associated enhancers^3^.

Evidence shows that most variants for common human diseases are not correlated with protein-coding changes, indicating that susceptibility variants in regulatory regions may contribute to disease phenotypes^16^. For CRC, most risk variants also reside outside the coding regions of genes^3^,^14^,^15^. Until now, as described above, comprehensive functional studies of CRC SNPs on nearby gene expression have been reported^3^,^14^,^15^. Evidence from the National Human Genome Research Institute (NHGRI) GWAS catalog shows that 85 CRC susceptibility variants, which reach suggestive association *P* < 1.00E-05, have been identified until now^17^,^18^. However, the exact genetic mechanisms for these newly identified CRC susceptibility variants are still unclear now. In order to investigate the potential regulatory functions for 85 newly identified CRC susceptibility variants, we conducted a pathway analysis of these CRC susceptibility genes around these CRC susceptibility variants.

## Results

### CRC susceptibility genes from ProxyGeneLD

Using the ProxyGeneLD and the LD information from the HapMap phase II Europe (CEU), 74 of these 85 unique CRC susceptibility variants were included in HapMap, and were successfully mapped to the corresponding genes 53 unique CRC susceptibility genes (Table 1). However, another 11 SNPs including rs11196172, rs73376930, rs11255841, rs10849432, rs35509282, rs140355816, rs34245511, rs12412391, rs4849303, rs57046232 and rs7999699 are not found in HapMap.

### CRC susceptibility genes for pathway analysis

Using the nearest upstream and downstream gene method in NHGRI GWAS catalog, we got 106 unique CRC susceptibility gene IDs as described above. We compared these 106 genes with 53 unique CRC susceptibility genes from the ProxyGeneLD, and found 31 shared genes. In the end, we got 128 unique CRC susceptibility genes, which is the union of genes from both methods.

### Pathway analysis preprocessing

In WebGestalt database, 120 of 128 genes were successfully mapped to 120 unique Entrez Gene IDs^19^. Other 8 genes were mapped to multiple Entrez Gene IDs or could not be mapped to any Entrez Gene ID. The following pathway analysis will be based upon the 120 unique gene IDs.

### Pathway analysis using GO database

Our pathway analysis in GO database showed that these 120 CRC susceptibility genes were significantly enriched in 40 biological processes, 1 molecular function and 3 cellular components with adjusted *P* < 0.01. 17 of these 44 significant signals are regulatory pathways, such as regulation of epithelial to mesenchymal transition, negative regulation of Wnt receptor signaling pathway, regulation of pathway-restricted SMAD protein phosphorylation, positive regulation of nucleocytoplasmic transport, regulation of muscle organ development, positive regulation of intracellular protein transport. Interestingly, regulation of epithelial to mesenchymal transition (GO:0010717) is the most significant signal (Table 2). More detailed information including the gene IDs is described in supplementary Table 1.

## Discussion

Until now, 85 CRC susceptibility variants with suggestive association *P* < 1.00E-05 have been reported^17^,^18^. To investigate the underlying genetic pathways where these newly identified CRC susceptibility genes are significantly enriched, we conducted a functional annotation. Using two kinds of SNP to gene mapping methods including the nearest upstream and downstream gene method and the ProxyGeneLD, we got 128 unique CRC susceptibility genes. We then conducted a pathway analysis in GO database using the corresponding 128 genes. We identified 44 GO categories, 17 of which are regulatory pathways.

Here, we identified the regulation of epithelial to mesenchymal transition (GO:0010717) to be the most significant signal in all the 44 GO categories and the most signal in all the 17 regulatory pathways. It is reported that the epithelial-mesenchymal transition-like dedifferentiation of the tumor cells is a character of CRC invasion^20^. Several studies have reviewed the association between epithelial-mesenchymal transition and CRC progression^21^,^22^,^23^. Our results show that these newly identified CRC susceptibility SNPs or genes may regulate epithelial-mesenchymal transition.

The negative regulation of Wnt receptor signaling pathway (GO:0030178) is the third significant signal in all the 44 GO categories and the second significant signal in all the 17 regulatory pathways. Evidence shows that aberrant regulation of the Wnt/β-catenin signaling pathway can cause CRC^24^. It is reported that the loss-of-function mutations in *APC* gene are common in CRC, and can cause inappropriate activation of Wnt signaling^24^. Recently, several studies have reviewed the involvement of Wnt signalling in CRC development^25^,^26^,^27^. Masuda *et al.* reported Wnt signaling to be the potential therapeutic target by targeting TNIK in CRC^28^. Here, our results show that these newly identified CRC susceptibility SNPs or genes may regulate Wnt receptor signaling pathway.

The positive regulation of nucleocytoplasmic transport pathway (GO:0046824) is the 8^th^ significant signal in all the 44 GO categories and the 4^th^ significant signal in all the 17 regulatory pathways. Hill *et al.* reviewed the mechanisms and role of nucleocytoplasmic transport in cancer therapy^29^. Here, we identified the pathway-restricted SMAD protein phosphorylation (GO:0060389) and regulation of pathway-restricted SMAD protein phosphorylation (GO:0060393) to be 5^th^ and 7^th^ significant association signals, respectively. Interestingly, evidence shows that protein phosphorylation is a post-translational modification central to cancer biology^30^. Protein phosphorylation affects most eukaryotic cellular processes and its deregulation is considered a hallmark of cancer^31^.

We also found that these newly identified CRC susceptibility SNPs or genes may regulate five GO categories related with cell differentiation including regulation of fat cell differentiation (GO:0045598), mesenchymal cell differentiation (GO:0048762), regulation of striated muscle cell differentiation (GO:0051153), negative regulation of myoblast differentiation (GO:0045662), and cell morphogenesis involved in differentiation (GO:0000904). Evidence showed the involvement of differentiation in CRC. Breaking the balance between proliferation and differentiation in animal cells can cause cancer^32^. PPAR-γ is a nuclear receptor with a dominant regulatory role in differentiation of cells of the adipose lineage^33^. PPAR-γ can modulate the growth and differentiation of CRC cells^33^. Differentiated human CRC cells protect tumor-initiating cells from irinotecan^34^. The resistance of colorectal tumors to irinotecan requires the cooperative action of tumor-initiating ALDHhigh/ABCB1negative cells and their differentiated, drug-expelling, ALDHlow/ABCB1positive daughter cells^34^. The calcium activated chloride channel A1 (CLCA1) is a member of the calcium sensitive chloride conductance family of proteins and is expressed mainly in the colon^32^. Recent study shows that CLCA1 plays an important role in differentiation and proliferation of Caco-2 cells, which can regulate the transition from proliferation to differentiation in CRC and may be a potential diagnostic marker for CRC prognosis^32^.

Take together, our findings suggest that most CRC susceptibility variants are located in the intron region of protein encoding genes and are not correlated with protein-coding changes. Most of these 120 CRC susceptibility genes are involved in kinds of regulatory pathways. Our results may provide further insight into the underlying genetic mechanisms for these newly identified CRC susceptibility variants.

## Materials and Methods

### CRC susceptibility variants

The CRC susceptibility variants were available from the NHGRI GWAS catalog, which collected the results of published GWAS in online database^18^. We selected 85 unique CRC susceptibility variants with *P* < 1.00E-05 from the GWAS CRC, CRC (calcium intake interaction), and CRC (diet interaction).

### Data preprocessing

In NHGRI GWAS catalog, these 85 unique CRC susceptibility variants were successfully mapped to 167 nearest upstream and downstream genes. We further analyzed these 167 genes and got 106 unique CRC susceptibility gene IDs. The detailed information was described in Table 1.

### Mapping SNPs to genes using the ProxyGeneLD

In addition to the nearest upstream and downstream gene method, we also used a Perl software named ProxyGeneLD. ProxyGeneLD can map these 85 SNPs to their corresponding genes using the linkage disequilibrium (LD) information from the HapMap genotyping data (HapMap phase II Europe (CEU), release 22)^35^. For more detailed algorithms, please refer to the original study^35^.

### CRC susceptibility genes

Here, we map these 85 SNPs to their corresponding genes using both methods as described above. The final CRC susceptibility gene set is the union of genes from both methods.

### Pathway analysis using WebGestalt

We used the GO pathways in WebGestalt database for pathway analysis^19^. The hypergeometric test was used to detect the overrepresentation of differently expressed AD genes among all of the genes in a given pathway^19^. The reference gene list is the entire Entrez gene set. The minimum number of genes for a category is 3. The FDR test was used to correct for multiple testing. GO pathways with an adjusted *P* < 0.05 are considered to be significantly associated with CRC.

## Additional Information

**How to cite this article**: Lu, X. *et al.* Colorectal cancer risk genes are functionally enriched in regulatory pathways. *Sci. Rep.* **6**, 25347; doi: 10.1038/srep25347 (2016).

## Supplementary Material

Supplementary Information

## Figures and Tables

**Table 1:** The main information for 85 CRC susceptibility variants.

SNP	Pubmed ID	Disease/Trait	Gene	*P* value	OR or beta	95% CI
rs4939827	18372901	CRC	SMAD7	8.00E-28	1.2	[1.16–1.24]
rs7014346	18372901	CRC	LOC101930033	9.00E-26	1.19	[1.15–1.23]
rs174537	24836286	CRC	MYRF	9.00E-21	1.16	[1.12–1.19]
rs16892766	18372905	CRC	LINC00536-EIF3H	3.00E-18	1.27	[1.20–1.34]
rs10795668	24836286	CRC	RNA5SP299-LINC00709	5.00E-15	1.15	[1.11–1.19]
rs6983267	17618284	CRC	CCAT2, LOC101930033	1.00E-14	1.27	[1.16–1.39]
rs647161	24836286	CRC	C5orf66	2.00E-14	1.15	[1.11–1.19]
rs6983267	24836286	CRC	CCAT2, LOC101930033	5.00E-14	1.14	[1.10–1.18]
rs10795668	18372905	CRC	RNA5SP299-LINC00709	3.00E-13	1.12	[1.10–1.16]
rs10505477	24737748	CRC	LOC101930033	8.00E-13	1.2	[NR]
rs4939827	17934461	CRC	SMAD7	1.00E-12	1.16	[1.09–1.27]
rs11196172	24836286	CRC	TCF7L2	1.00E-12	1.14	[1.10–1.18]
rs2423279	24836286	CRC	SRSF10P2	3.00E-12	1.13	[1.09–1.17]
rs73376930	24737748	CRC	GREM1, LOC100131315	1.00E-11	1.25	[NR]
rs6983267	23266556	CRC	CCAT2, LOC101930033	1.00E-11	1.13	[1.09–1.18]
rs10505477	17618283	CRC	LOC101930033	3.00E-11	1.17	[1.12–1.23]
rs7229639	24448986	CRC	SMAD7	3.00E-11	1.22	[1.15–1.29]
rs2427308	24737748	CRC	CABLES2	3.00E-11	1.24	[NR]
rs1035209	24737748	CRC	NKX2-3-SLC25A28	5.00E-11	1.12	[1.08–1.16]
rs6983267	18372905	CRC	CCAT2, LOC101930033	7.00E-11	1.24	[1.17–1.33]
rs11255841	24737748	CRC	RNA5SP299-LINC00709	7.00E-11	1.19	[NR]
rs1321311	22634755	CRC	N/A	1.00E-10	1.1	[1.07–1.13]
rs10774214	24836286	CRC	RPL18P9-CCND2	1.00E-10	1.14	[1.09–1.18]
rs647161	23263487	CRC	C5orf66	1.00E-10	1.11	[1.08–1.15]
rs961253	19011631	CRC	FGFR3P3-CASC20	2.00E-10	1.12	[1.08–1.16]
rs11169552	20972440	CRC	DIP2B-ATF1	2.00E-10	1.09	[1.05–1.11]
rs4925386	20972440	CRC	LAMA5	2.00E-10	1.08	[1.05–1.10]
rs4939827	23266556	CRC	SMAD7	2.00E-10	1.12	[1.09–1.16]
rs3824999	22634755	CRC	POLD3	4.00E-10	1.08	[1.05–1.10]
rs647161	23263487	CRC	C5orf66	4.00E-10	1.17	[1.11–1.22]
rs10774214	23263487	CRC	RPL18P9-CCND2	5.00E-10	1.17	[1.11–1.23]
rs3802842	18372901	CRC	COLCA2, COLCA1	6.00E-10	1.11	[1.08–1.15]
rs10849432	24836286	CRC	LOC102723767	6.00E-10	1.14	[1.09–1.18]
rs5934683	22634755	CRC	GPR143-SHROOM2	7.00E-10	1.07	[1.04–1.10]
rs4444235	19011631	CRC	RPS3AP46-MIR5580	8.00E-10	1.11	[1.08–1.15]
rs6691170	20972440	CRC	DUSP10-QRSL1P2	1.00E-09	1.06	[1.03–1.09]
rs12241008	25105248	CRC	VTI1A	1.00E-09	1.13	[1.09–1.18]
rs6687758	20972440	CRC	DUSP10-QRSL1P2	2.00E-09	1.09	[1.06–1.12]
rs10411210	19011631	CRC	RHPN2	5.00E-09	1.15	[1.10–1.20]
rs2423279	23263487	CRC	SRSF10P2	7.00E-09	1.1	[1.06–1.14]
rs35509282	25023989	CRC	MTHFD2P4-TOMM22P4	8.00E-09	1.53	[1.39–1.67]
rs7758229	21242260	CRC	SLC22A3	8.00E-09	1.28	[1.18–1.39]
rs6687758	24836286	CRC	DUSP10-QRSL1P2	9.00E-09	1.12	[1.08–1.17]
rs4143094	24743840	CRC^b^	GATA3	9.00E-09	1.17	[1.11–1.23]
rs9929218	19011631	CRC	CDH1	1.00E-08	1.1	[1.06–1.12]
rs1800469	24836286	CRC	TGFB1, B9D2	1.00E-08	1.09	[1.06–1.12]
rs4779584	21761138	CRC	SCG5-GREM1	2.00E-08	1.18	[1.11–1.24]
rs704017	24836286	CRC	ZMIZ1-AS1	2.00E-08	1.1	[1.06–1.13]
rs7229639	24836286	CRC	SMAD7	2.00E-08	1.2	[1.16–1.25]
rs140355816	24737748	CRC	LINC00536-EIF3H	2.00E-08	1.59	[NR]
rs6983267	21242260	CRC	CCAT2, LOC101930033	2.00E-08	1.18	[1.11–1.25]
rs10936599	20972440	CRC	MYNN	3.00E-08	1.04	[1.04–1.10]
rs12603526	24836286	CRC	NXN	3.00E-08	1.1	[1.06–1.14]
rs10774214	23263487	CRC	RPL18P9-CCND2	3.00E-08	1.09	[1.06–1.13]
rs34245511	24737748	CRC	LIMA1	3.00E-08	1.14	[NR]
rs367615	23300701	CRC	KRT18P42-MAN2A1	4.00E-08	1.35	[1.20–1.49]
rs11903757	23266556	CRC	NABP1-SDPR	4.00E-08	1.16	[1.10–1.22]
rs6469656	24836286	CRC	LINC00536-EIF3H	5.00E-08	1.09	[1.06–1.13]
rs3217810	23266556	CRC	CCND2	6.00E-08	1.2	[1.12–1.28]
rs4948317	24836286	CRC	BICC1	7.00E-08	1.1	[1.06–1.13]
rs13343954	24737748	CRC	RHPN2	7.00E-08	1.18	[NR]
rs10911251	23266556	CRC	LAMC1	9.00E-08	1.09	[1.06–1.13]
rs4939827	21761138	CRC	SMAD7	1.00E-07	1.14	[1.08–1.18]
rs2423279	23263487	CRC	SRSF10P2	2.00E-07	1.14	[1.08–1.19]
rs3802842	24836286	CRC	COLCA2, COLCA1	3.00E-07	1.09	[1.05–1.12]
rs3217901	23266556	CRC	CCND2	3.00E-07	1.1	[1.06–1.14]
rs13130787	23266556	CRC	ATOH1-HMGB3P15	3.00E-07	1.09	[1.06–1.13]
rs16892766	21761138	CRC	LINC00536-EIF3H	4.00E-07	1.24	[1.14–1.34]
rs3802842	21761138	CRC	COLCA2, COLCA1	4.00E-07	1.14	[1.08–1.20]
rs3802842	23266556	CRC	COLCA2, COLCA1	4.00E-07	1.11	[1.06–1.15]
rs59336	23266556	CRC	TBX3	4.00E-07	1.09	[1.06–1.13]
rs4779584	18372905	CRC	SCG5-GREM1	5.00E-07	1.23	[1.14–1.34]
rs4779584	23266556	CRC	SCG5-GREM1	5.00E-07	1.12	[1.08–1.19]
rs12412391	24836286	CRC	LOC101927324, LINC01475	7.00E-07	1.08	[1.05–1.11]
rs1028166	25192705	CRC^a^	N/A	7.00E-07	1.49	[1.27–1.74]
rs2901879	24743840	CRC^b^	MEIS1-AS2-DNMT3AP1	7.00E-07	1.11	[1.06–1.16]
rs1871438	24743840	CRC^b^	NPM1P5-ST8SIA2	8.00E-07	1.11	[1.07–1.16]
rs1665650	23263487	CRC	HSPA12A	9.00E-07	1.13	[1.08–1.19]
rs4849303	24743840	CRC^b^	ACOXL	1.00E-06	1.12	[1.08–1.18]
rs4147045	24743840	CRC^b^	KRT18P63-RPL21P46	1.00E-06	1.2	[1.11–1.3]
rs8180040	23350875	CRC	KLHL18-PTPN23	2.00E-06	1.28	[1.15–1.41]
rs4939827	18372905	CRC	SMAD7	2.00E-06	1.18	[1.10–1.25]
rs39453	23300701	CRC	SNRPCP19-CYCS	2.00E-06	1.28	[1.16–1.43]
rs6855885	25192705	CRC^a^	CCSER1	2.00E-06	1.11	[1.06–1.16]
rs1933755	25192705	CRC^a^	TMEM200A-SMLR1	2.00E-06	1.19	[1.11–1.28]
rs1370916	24743840	CRC^b^	MRPL42P4-TNS3	2.00E-06	1.11	[1.06–1.15]
rs6989010	24743840	CRC^b^	LINC00681-KIAA1456	2.00E-06	1.64	[1.33–2]
rs2593957	24743840	CRC^b^	MORC1	2.00E-06	1.15	[1.09–1.2]
rs12534701	24743840	CRC^b^	DPP6	2.00E-06	1.17	[1.09–1.24]
rs4855695	24743840	CRC^b^	MORC1-FLJ22763	2.00E-06	1.14	[1.08–1.20]
rs12548021	23350875	CRC	DUSP4-RPL17P33	3.00E-06	1.28	[1.155–1.42]
rs12309274	24836286	CRC	WNK1	3.00E-06	1.11	[1.06–1.16]
rs10411210	24836286	CRC	RHPN2	3.00E-06	1.12	[1.07–1.17]
rs17730929	23300701	CRC	RPS23P3-CENPC	3.00E-06	1.47	[1.25–1.72]
rs10114408	23300701	CRC	MIR4291-BARX1	3.00E-06	1.37	[1.20–1.56]
rs4591517	23300701	CRC	SALL4P5-RPL24P7	3.00E-06	1.06	[0.88–1.29]
rs10879357	23300701	CRC	TPH2	3.00E-06	1.25	[1.14–1.39]
rs57046232	24737748	CRC	FGFR3P3-CASC20	3.00E-06	1.11	[NR]
rs2057314	23266556	CRC	DCBLD1	3.00E-06	1.08	[1.04–1.11]
rs17094983	23266556	CRC	DACT1-RPL31P4	3.00E-06	1.13	[1.08–1.20]
rs7999699	24743840	CRC^b^	RPL27AP8-SUCLA2	3.00E-06	1.32	[1.18–1.47]
rs3104964	23350875	CRC	LOC100616530, C8orf37-AS1	4.00E-06	1.27	[1.14–1.40]
rs9365723	23300701	CRC	SYNJ2	4.00E-06	1.27	[1.15–1.41]
rs11671104	24743840	CRC^b^	GOLGA2P9-ZNF492	4.00E-06	1.25	[1.13–1.37]
rs10752881	24836286	CRC	KRT18P28-LAMC1	5.00E-06	1.07	[1.04–1.10]
rs7248888	24743840	CRC^b^	PNMAL1	5.00E-06	1.37	[1.19–1.56]
rs7315438	21761138	CRC	TBX3-UBA52P7	6.00E-06	1.11	[1.06–1.15]
rs4813802	23266556	CRC	CASC20-BMP2	7.00E-06	1.1	[1.05–1.14]
rs2128382	23266556	CRC	GSDMC-FAM49B	8.00E-06	1.11	[1.06–1.16]
rs1912453	23266556	CRC	C1orf110	9.00E-06	1.07	[1.04–1.11]

CRC, colorectal cancer; CRC^a^, CRC (calcium intake interaction); CRC^b^, CRC (diet interaction); OR, odds ratio; CI, confidence interval; Chr, Chromosome; NR, not reported.

**Table 2:** Significant GO pathways from pathway analysis of 128 CRC susceptibility genes.

GO categories	ID	Name	C	O	E	R	rawP	adjP
biological process	GO:0010717	regulation of epithelial to mesenchymal transition	38	5	0.17	28.73	7.73E-07	8.55E-05
biological process	GO:0060675	ureteric bud morphogenesis	68	6	0.31	19.27	6.43E-07	8.55E-05
biological process	GO:0030178	negative regulation of Wnt receptor signaling pathway	112	7	0.51	13.65	7.60E-07	8.55E-05
biological process	GO:0035295	tube development	436	12	2	6.01	5.73E-07	8.55E-05
biological process	GO:0060389	pathway-restricted SMAD protein phosphorylation	37	5	0.17	29.51	6.74E-07	8.55E-05
biological process	GO:0001657	ureteric bud development	104	7	0.48	14.7	4.59E-07	8.55E-05
biological process	GO:0060393	regulation of pathway-restricted SMAD protein phosphorylation	33	5	0.15	33.08	3.72E-07	8.55E-05
biological process	GO:0046824	positive regulation of nucleocytoplasmic transport	73	6	0.33	17.95	9.82E-07	9.50E-05
biological process	GO:0048468	cell development	1461	21	6.69	3.14	1.20E-06	1.00E-04
biological process	GO:0048634	regulation of muscle organ development	104	6	0.48	12.6	7.84E-06	2.00E-04
biological process	GO:0090316	positive regulation of intracellular protein transport	88	6	0.4	14.89	2.96E-06	2.00E-04
biological process	GO:0032989	cellular component morphogenesis	1000	16	4.58	3.49	8.23E-06	2.00E-04
biological process	GO:0048729	tissue morphogenesis	472	11	2.16	5.09	8.81E-06	2.00E-04
biological process	GO:0048869	cellular developmental process	2829	29	12.96	2.24	6.09E-06	2.00E-04
biological process	GO:0051222	positive regulation of protein transport	163	7	0.75	9.38	9.29E-06	2.00E-04
biological process	GO:0001658	branching involved in ureteric bud morphogenesis	64	5	0.29	17.06	1.07E-05	2.00E-04
biological process	GO:0061138	morphogenesis of a branching epithelium	169	7	0.77	9.04	1.18E-05	2.00E-04
biological process	GO:0009887	organ morphogenesis	802	15	3.67	4.08	2.53E-06	2.00E-04
biological process	GO:0090092	regulation of transmembrane receptor protein serine/threonine kinase signaling pathway	168	7	0.77	9.1	1.13E-05	2.00E-04
biological process	GO:0045598	regulation of fat cell differentiation	64	5	0.29	17.06	1.07E-05	2.00E-04
biological process	GO:0016202	regulation of striated muscle tissue development	102	6	0.47	12.84	7.01E-06	2.00E-04
biological process	GO:0060485	mesenchyme development	162	7	0.74	9.44	8.93E-06	2.00E-04
biological process	GO:0042476	odontogenesis	103	6	0.47	12.72	7.41E-06	2.00E-04
biological process	GO:0035239	tube morphogenesis	300	9	1.37	6.55	8.57E-06	2.00E-04
biological process	GO:0022612	gland morphogenesis	110	6	0.5	11.91	1.08E-05	2.00E-04
biological process	GO:0048732	gland development	277	9	1.27	7.09	4.49E-06	2.00E-04
biological process	GO:0048762	mesenchymal cell differentiation	141	7	0.65	10.84	3.57E-06	2.00E-04
biological process	GO:0032388	positive regulation of intracellular transport	95	6	0.44	13.79	4.64E-06	2.00E-04
biological process	GO:0002009	morphogenesis of an epithelium	370	10	1.69	5.9	6.51E-06	2.00E-04
biological process	GO:2000027	regulation of organ morphogenesis	147	7	0.67	10.4	4.71E-06	2.00E-04
biological process	GO:0034330	cell junction organization	195	8	0.89	8.96	2.87E-06	2.00E-04
biological process	GO:0051153	regulation of striated muscle cell differentiation	64	5	0.29	17.06	1.07E-05	2.00E-04
biological process	GO:0042307	positive regulation of protein import into nucleus	61	5	0.28	17.9	8.45E-06	2.00E-04
biological process	GO:0045662	negative regulation of myoblast differentiation	10	3	0.05	65.51	1.08E-05	2.00E-04
biological process	GO:0001763	morphogenesis of a branching structure	196	8	0.9	8.91	2.98E-06	2.00E-04
biological process	GO:0000902	cell morphogenesis	945	16	4.33	3.7	4.00E-06	2.00E-04
biological process	GO:0048646	anatomical structure formation involved in morphogenesis	1594	21	7.3	2.88	4.88E-06	2.00E-04
biological process	GO:0000904	cell morphogenesis involved in differentiation	704	13	3.22	4.03	1.52E-05	3.00E-04
biological process	GO:0060284	regulation of cell development	495	11	2.27	4.85	1.38E-05	3.00E-04
biological process	GO:1901213	regulation of transcription from RNA polymerase II promoter involved in heart development	12	3	0.05	54.59	1.96E-05	3.00E-04
molecular function	GO:0008013	beta-catenin binding	63	5	0.28	18.1	8.03E-06	6.00E-04
cellular component	GO:0005913	cell-cell adherens junction	46	4	0.19	21.18	3.73E-05	3.30E-03
cellular component	GO:0070161	anchoring junction	218	6	0.89	6.7	3.00E-04	8.80E-03
cellular component	GO:0005912	adherens junction	201	6	0.83	7.27	2.00E-04	8.80E-03

C, the number of reference genes in the category; O, the number of genes in the gene set and also in the category; E, expected number in the category; R, the ratio of enrichment, rawP, the p value from hypergeometric test; adjP, the p value adjusted by the multiple test adjustment.
