# Supplementary material for: Colorectal cancer risk genes are functionally enriched in regulatory pathways
Source: Sci Rep. 2016 May 5;6:25347. doi: 10.1038/srep25347 (PMC4857176; doi:10.1038/srep25347)
Supplement: Supplementary Information [file srep25347-s1.pdf]

Supplementary Information

Colorectal cancer risk genes are functionally enriched in regulatory pathways

Xi Lu[a], Mingming Cao[b], Su Han[c], Youlin Yang[a, *], Jin Zhou[d, *]

[a]Department of Gastroenterology, The First Affiliated Hospital of Harbin Medical University, No. 23 You zheng Street Nan Gang District, Harbin, Heilongjiang 150001, China.

[b]Department of Endocrinology, The First Affiliated Hospital of Harbin Medical University, No. 23 You zheng Street Nan Gang District, Harbin, Heilongjiang 150001, China.

[c]Department of Parasitology, Harbin Medical University, Harbin, Heilongjiang 150081, China

[d]Department of Hematology, The First Affiliated Hospital of Harbin Medical University, No. 23 You zheng Street Nan Gang District, Harbin, Heilongjiang 150001, China.

*Corresponding author: Jin Zhou

Department of Hematology, The First Affiliated Hospital of Harbin Medical University, No. 23 You zheng Street Nan Gang District, Harbin, Heilongjiang 150001, China.

E-mail: jinzhou0451@163.com

*Corresponding author: Youlin Yang

[a]Department of Gastroenterology, The First Affiliated Hospital of Harbin Medical University, No. 23 You zheng Street Nan Gang District, Harbin, Heilongjiang 150001, China.

E-mail: youlinyanghh@163.com

Supplementary Table 1, significant GO pathways from pathway analysis of 128 CRC susceptibility genes

GO categories	Pathway ID	Pathway Name	Gene
biological process	GO:0010717	regulation of epithelial to mesenchymal transition	7040 650 26585 6934 4092
biological process	GO:0060675	ureteric bud morphogenesis	7040 650 2625 26585 652 3911
biological process	GO:0030178	negative regulation of Wnt receptor signaling pathway	64359 650 26585 56033 51339 6934 80114
biological process	GO:0035295	tube development	7040 650 26585 64759 999 6926 29072 4092 4124 2625 652 3911
biological process	GO:0060389	pathway-restricted SMAD protein phosphorylation	7040 650 26585 652 4092
biological process	GO:0001657	ureteric bud development	7040 650 2625 26585 652 3911 4092
biological process	GO:0060393	regulation of pathway-restricted SMAD protein phosphorylation	7040 650 26585 652 4092
biological process	GO:0046824	positive regulation of nucleocytoplasmic transport	7040 26585 652 999 51339 6934
biological process	GO:0048468	cell development	7040 26585 3915 29072 4092 2625 474 23218 6934 650 466 65125 999 51339 6926 11221 652 745 894 8128 3911
biological process	GO:0048634	regulation of muscle organ development	7040 650 26585 652 6926 6934
biological process	GO:0090316	positive regulation of intracellular protein transport	7040 26585 652 999 51339 6934

biological process	GO:0032989	cellular component morphogenesis	7040 650 26585 3915 80776 51339 4092 25930 2625 652 474 8128 23218 357 3911 6934
biological process	GO:0048729	tissue morphogenesis	7040 650 26585 999 51339 6926 29072 4092 2625 652 3911
biological process	GO:0048869	cellular developmental process	3992 7040 26585 3915 80776 29072 4092 25930 64359 2625 474 23218 27136 6934 650 466 65125 999 51339 6926 159296 11221 652 745 894 8128 357 56033 3911
biological process	GO:0051222	positive regulation of protein transport	7040 2625 26585 652 999 51339 6934
biological process	GO:0001658	branching involved in ureteric bud morphogenesis	7040 650 26585 652 3911
biological process	GO:0061138	morphogenesis of a branching epithelium	7040 650 26585 652 999 3911 6926
biological process	GO:0009887	organ morphogenesis	7040 650 26585 999 51339 6926 29072 159296 4092 2625 652 474 357 3911 6934
biological process	GO:0090092	regulation of transmembrane receptor protein serine/threonine kinase signaling pathway	7040 650 26585 652 51339 6934 4092
biological process	GO:0045598	regulation of fat cell differentiation	7040 650 2625 51339 6934
biological process	GO:0016202	regulation of striated muscle tissue development	7040 650 26585 652 6926 6934
biological process	GO:0060485	mesenchyme development	7040 650 26585 652 3911 6934 4092

biological process	GO:0042476	odontogenesis	7040 650 652 3911 6934 159296
biological process	GO:0035239	tube morphogenesis	7040 650 26585 999 6926 29072 2625 652 3911
biological process	GO:0022612	gland morphogenesis	7040 652 999 3911 6926 159296
biological process	GO:0048732	gland development	7040 650 999 6926 159296 2625 652 3911 6934
biological process	GO:0048762	mesenchymal cell differentiation	7040 650 26585 652 3911 6934 4092
biological process	GO:0032388	positive regulation of intracellular transport	7040 26585 652 999 51339 6934
biological process	GO:0002009	morphogenesis of an epithelium	7040 650 26585 999 51339 6926 29072 2625 652 3911
biological process	GO:2000027	regulation of organ morphogenesis	7040 650 2625 26585 652 999 51339
biological process	GO:0034330	cell junction organization	7040 1001 26585 3915 999 357 3911 4092
biological process	GO:0051153	regulation of striated muscle cell differentiation	7040 650 26585 652 6926
biological process	GO:0042307	positive regulation of protein import into nucleus	7040 26585 652 999 51339
biological process	GO:0045662	negative regulation of myoblast differentiation	7040 652 6926
biological	GO:0001763	morphogenesis of a branching structure	7040 650 26585 652 999 3911 6926 29072

process			
biological process	GO:0000902	cell morphogenesis	7040 650 26585 3915 80776 51339 4092 25930 2625 652 474 8128 23218 357 3911 6934
biological process	GO:0048646	anatomical structure formation involved in morphogenesis	7040 26585 3915 80776 29072 4092 25930 2625 474 23218 1846 6934 650 999 51339 6926 159296 652 8128 357 3911
biological process	GO:0000904	cell morphogenesis involved in differentiation	7040 650 26585 3915 51339 4092 2625 652 474 23218 8128 3911 6934
biological process	GO:0060284	regulation of cell development	7040 650 26585 466 6926 4092 11221 652 474 894 6934
biological process	GO:1901213	regulation of transcription from RNA polymerase II promoter involved in heart development	650 26585 652
molecular function	GO:0008013	beta-catenin binding	999 357 51339 6934 4092
cellular component	GO:0005913	cell-cell adherens junction	1001 999 357 4092
cellular component	GO:0070161	anchoring junction	1001 64759 999 357 51474 4092
cellular component	GO:0005912	adherens junction	1001 64759 999 357 51474 4092
